# AI-based dimensional neuroimaging system for characterizing heterogeneity in brain structure and function in major depressive disorder: COORDINATE-MDD consortium design and rationale

**DOI:** 10.1186/s12888-022-04509-7

**Published:** 2023-01-23

**Authors:** Cynthia H. Y. Fu, Guray Erus, Yong Fan, Mathilde Antoniades, Danilo Arnone, Stephen R. Arnott, Taolin Chen, Ki Sueng Choi, Cherise Chin Fatt, Benicio N. Frey, Vibe G. Frokjaer, Melanie Ganz, Jose Garcia, Beata R. Godlewska, Stefanie Hassel, Keith Ho, Andrew M. McIntosh, Kun Qin, Susan Rotzinger, Matthew D. Sacchet, Jonathan Savitz, Haochang Shou, Ashish Singh, Aleks Stolicyn, Irina Strigo, Stephen C. Strother, Duygu Tosun, Teresa A. Victor, Dongtao Wei, Toby Wise, Rachel D. Woodham, Roland Zahn, Ian M. Anderson, J. F. William Deakin, Boadie W. Dunlop, Rebecca Elliott, Qiyong Gong, Ian H. Gotlib, Catherine J. Harmer, Sidney H. Kennedy, Gitte M. Knudsen, Helen S. Mayberg, Martin P. Paulus, Jiang Qiu, Madhukar H. Trivedi, Heather C. Whalley, Chao-Gan Yan, Allan H. Young, Christos Davatzikos

**Affiliations:** 1 Department of Psychological Sciences, University of East London, London, UK; 2 Centre for Affective Disorders, Department of Psychological Medicine, Institute of Psychiatry, Psychology, and Neuroscience, King’s College London, London, UK; 3 Center for Biomedical Image Computing and Analytics, Perelman School of Medicine, University of Pennsylvania, Philadelphia, USA; 4 Department of Psychiatry and Behavioral Science, College of Medicine and Health Sciences, United Arab Emirates University, Al Ain, United Arab Emirates; 5 Rotman Research Institute, Baycrest Centre, Toronto, Canada; 6 Huaxi MR Research Center, Department of Radiology, West China Hospital, Sichuan University, Chengdu, China; 7 Research Unit of Psychoradiology, Chinese Academy of Medical Sciences, Chengdu, China; 8 Nash Family Center for Advanced Circuit Therapeutics, Icahn School of Medicine at Mount Sinai, New York, USA; 9 Department of Psychiatry, Center for Depression Research and Clinical Care, University of Texas Southwestern Medical Center, Dallas, USA; 10 Department of Psychiatry and Behavioural Neurosciences, McMaster University, Hamilton, Canada; 11 Mood Disorders Treatment and Research Centre and Women’s Health Concerns Clinic, St Joseph’s Healthcare Hamilton, Hamilton, Canada; 12 Neurobiology Research Unit, University Hospital Rigshospitalet, Copenhagen, Denmark; 13 Department of Clinical Medicine, Faculty of Health and Medical Sciences, University of Copenhagen, Copenhagen, Denmark; 14 Department of Psychiatry, Psychiatric Centre Copenhagen, Copenhagen, Denmark; 15 Department of Computer Science, University of Copenhagen, Copenhagen, Denmark; 16 Department of Psychiatry, University of Oxford, Oxford, UK; 17 Oxford Health NHS Foundation Trust, Warneford Hospital, Oxford, UK; 18 Mathison Centre for Mental Health Research and Education, University of Calgary, Calgary, Canada; 19 Department of Psychiatry, Cumming School of Medicine, University of Calgary, Calgary, Canada; 20 Department of Psychiatry, University Health Network, Toronto, Canada; 21 Division of Psychiatry, Royal Edinburgh Hospital, University of Edinburgh, Edinburgh, UK; 22 Centre for Depression and Suicide Studies, Unity Health Toronto, Toronto, Canada; 23 Meditation Research Program, Department of Psychiatry, Massachusetts General Hospital, Harvard Medical School, Boston, USA; 24 Laureate Institute for Brain Research, Tulsa, USA; 25 Penn Statistics in Imaging and Visualization Endeavor (PennSIVE) Center, Department of Biostatistics, Epidemiology and Informatics, University of Pennsylvania, Philadelphia, USA; 26 Department of Radiology and Biomedical Imaging, University of California San Francisco, San Francisco, USA; 27 Department of Medical Biophysics, University of Toronto, Toronto, Canada; 28 School of Psychology, Southwest University, Chongqing, China; 29 Department of Neuroimaging, Institute of Psychiatry, Psychology and Neuroscience, King’s College London, London, UK; 30 Division of Neuroscience and Experimental Psychology, University of Manchester, Manchester, UK; 31 Department of Psychiatry and Behavioral Sciences, Emory University School of Medicine, Atlanta, USA; 32 Department of Psychology, Stanford University, Stanford, USA; 33 Unity Health Toronto, Toronto, Canada; 34 CAS Key Laboratory of Behavioral Science, Institute of Psychology, Beijing, China; 35 South London and Maudsley NHS Foundation Trust, Bethlem Royal Hospital, London, UK

**Keywords:** Classification, Biomarkers, Deep learning, Neuroimaging, Depression, Harmonization, Predictors, MRI

## Abstract

**Background:**

Efforts to develop neuroimaging-based biomarkers in major depressive disorder (MDD), at the individual level, have been limited to date. As diagnostic criteria are currently symptom-based, MDD is conceptualized as a disorder rather than a disease with a known etiology; further, neural measures are often confounded by medication status and heterogeneous symptom states.

**Methods:**

We describe a consortium to quantify neuroanatomical and neurofunctional heterogeneity via the dimensions of novel multivariate coordinate system (COORDINATE-MDD). Utilizing imaging harmonization and machine learning methods in a large cohort of medication-free, deeply phenotyped MDD participants, patterns of brain alteration are defined in replicable and neurobiologically-based dimensions and offer the potential to predict treatment response at the individual level.

International datasets are being shared from multi-ethnic community populations, first episode and recurrent MDD, which are medication-free, in a current depressive episode with prospective longitudinal treatment outcomes and in remission. Neuroimaging data consist of de-identified, individual, structural MRI and resting-state functional MRI with additional positron emission tomography (PET) data at specific sites. State-of-the-art analytic methods include automated image processing for extraction of anatomical and functional imaging variables, statistical harmonization of imaging variables to account for site and scanner variations, and semi-supervised machine learning methods that identify dominant patterns associated with MDD from neural structure and function in healthy participants.

**Results:**

We are applying an iterative process by defining the neural dimensions that characterise deeply phenotyped samples and then testing the dimensions in novel samples to assess specificity and reliability. Crucially, we aim to use machine learning methods to identify novel predictors of treatment response based on prospective longitudinal treatment outcome data, and we can externally validate the dimensions in fully independent sites.

**Conclusion:**

We describe the consortium, imaging protocols and analytics using preliminary results. Our findings thus far demonstrate how datasets across many sites can be harmonized and constructively pooled to enable execution of this large-scale project.

## Background

Depression has been recognized for millennia as a distinct illness, included in what Hippocrates termed ‘melancholia’ and posited to be caused by black bile. Current diagnostic criteria for major depressive disorder (MDD) are based solely on a set of symptoms and observable behaviors. MDD is characterised by a persistent low mood and/or an inability to experience usual feelings of enjoyment, associated with disturbances in sleep, appetite and psychomotor functioning, low energy, poor concentration, guilt or worthlessness, and, for some, suicidal ideation and behaviors [1, 2]. MDD is highly prevalent and has significant personal, familial and socioeconomic impacts [3, 4]. At the present time, MDD remains a syndrome without an identified etiology, rather than a disease with a demonstrable pathology. There are no neurobiological markers that can identify the diseases which comprise a clinical MDD diagnosis. We lack reproducible neurobiological markers to improve the etiological and prognostic homogeneity as well as to predict response to treatment.

Not only can heterogeneous combinations of symptoms fulfil diagnostic criteria, but current criteria do not fully capture the range of symptoms. For example, high levels of anxiety and comorbid anxiety disorders are present in 50–75% of MDD individuals, which are further linked with impaired treatment response and chronic longitudinal course [5, 6]. Heterogeneity among clinical profiles can lead to less predictable responses to a given treatment, an inability to predict the longitudinal course for individual patients, and the symptom variability in ‘gold standard’ scales designated to evaluate efficacy of therapeutic interventions. Further, treatment outcomes are frequently unsatisfactory. For 30–40% of MDD individuals in a current depressive episode, an adequate treatment response or remission is not achieved even after several trials of pharmacotherapy or psychotherapy over a year [7, 8].

It is unlikely that MDD is caused by a single etiological factor. MDD heritability estimates range from 28 to 44% [9] and are considerably lower than estimates for bipolar disorder and schizophrenia, which range from 60 to 90% [10, 11]. Genome-wide association and candidate gene studies have powerfully demonstrated polygenic heritability, consisting of hundreds of variants and genes with each having a small genetic contribution. However, genetic risk variants have not clinically useful at the individual level for diagnosis, and it is unclear how genetic risk ultimately translates into an acute depressive episode [12].

Neuroimaging-based biomarkers can help to identify the various disease components that comprise MDD. Genetic and environmental factors that lead to MDD are expressed as subtle and widespread alterations in brain structure and brain function. Research over two decades provides convincing evidence of morphometric and neural alterations in MDD, despite limitations in diagnosis and treatment selection. An overarching aim has been to delineate the neurobiological features that comprise MDD and to develop imaging markers in this disorder [13–15]. It is important to recognize though that to search for a neural signature that wholly replicates current diagnostic criteria would be circular, *petitio principii*, because current diagnostic criteria are based on clinical features and different mechanisms could lead to the same clinical presentation. Efforts have been further hindered by neural measures which have been confounded by a mixture of depressive states, multiple and longstanding treatments, as well as comorbid disorders.

In recent years, machine learning based methods for MRI heterogeneity analysis have been developed which detect and characterize neuroanatomical heterogeneity of disease using a data-driven approach that generates quantifiable, replicable and neurobiologically-based metrics of disease subtypes [16]. Large samples have recently been created from multi-site datasets, however the multivariate pattern analysis has been limited to either resting-state functional MRI data or structural MRI data with limited clinical phenotyping and lack of longitudinal treatment outcome data. From resting-state functional MRI data, four functional connectivity patterns were observed in frontostriatal and limbic systems which reflected different symptom profiles [17]. However, the training and cross-validation samples were confounded by medication and form of depression, namely treatment-resistant depression, which has demonstrable effects on brain structure and function [18–20], and there was no independent testing in a novel sample. Moreover, the models were not reproduced in an independent study [21], which could reflect overfitting in the clustering algorithm as well as insufficient overlap in the sample characteristics. Resting state functional MRI reflects depressive state, and these samples had distinct depressive states, multiple comorbid disorders, different forms of depression, with neurofunctional correlates that were likely underpowered for such non-overlapping samples [17–21].

In structural MRI, recent multi-site cohorts show classification accuracies ranging from 52 to 75% [22–25]. However, all the dimensions have been binary (ie. either MDD or healthy control). The highest classification accuracy was achieved in a cohort with a MDD diagnosis based on diagnostic criteria and that was in a current depressive episode, but the sample size was limited (230 MDD, 77 controls) [22]. In the largest sample to date (2288 MDD and 3077 controls), the Enhancing Neuro Imaging Genetics through Meta-Analysis (ENIGMA) consortium found a classification accuracy up to 62% [23]. However, the ENIGMA MDD sample consists of a wide range of clinical phenotypes, with multiple comorbid disorders, many forms of depression from first episode to treatment-resistant depression, but limited medication history, antidepressant dosage or duration, and no treatment outcome data [26]. Similarly, large samples from UK Biobank data have limited treatment history, no treatment outcome data, and are based on a probable diagnosis of lifetime MDD derived from self-report symptoms in a population-based sample [27].

Our consortium aims to identify imaging signatures of disease heterogeneity in MDD using structural and resting state functional MRI. This will generate a neuroanatomical-neurofunctional (NA-NF) dimensional coordinate system (COORDINATE-MDD) in which each dimension captures a distinct pattern of brain alterations. Our aim is to identify the multivariate dimensions that define disease-related phenotypes in MDD and the distinct dimensions that predict treatment response at the individual level. Importantly, this is an iterative process to: (i) define neural dimensions in deeply phenotyped participants who are medication-free and in a current depressive episode in order to delineate state and trait status; and (ii) test dimensions in novel samples to assess specificity and reproducibility. With this aim, our consortium combines extensive datasets of ‘raw’ individual-level neuroimaging and deeply phenotyped clinical data, using state-of-the-art analytic methods for big data and semi-supervised clustering. The present sample consists of richly phenotyped, individual-level data from participants with first episode or recurrent MDD, that is not treatment-resistant depression, antidepressant medication-free, with prospective longitudinal treatment outcomes, and healthy controls. The current focus is on structural and resting-state functional MRI.

We have sought to focus on first episode and recurrent MDD in the present sample. Treatment-resistant depression is currently a clinical criterion that refers to a form of depression which shows significant persistent symptoms despite a series of treatments. If treatment resistance is present at the first episode, then it might be possible to identify this dimension early in the illness. Low rates of remission associated with current treatments demonstrate their limitations [28] and could also indicate a subgroup that will progress to fulfil the clinical criterion of treatment resistance. It is possible that the pathophysiology of treatment-resistant depression might be characterized early in the course of illness [14].

Sample size directly influences the capacity of machine learning methods to reliably identify imaging signatures of disease from MRI data and machine learning-based signatures will need to be replicated in independent cohorts. Through international collaborations, we are bringing together a large and integrated sample. MRI images are processed using image processing methods that leverage robust and fully automated pipelines for extracting structural and functional imaging features. In large multi-site datasets, harmonization of imaging features from each site is a critical requirement. We will apply a statistical harmonization methodology, Combat-GAM [29], developed for pooling neuroimaging data across multiple scanners and cohorts with diverse age ranges and with the presence of nonlinear age-related differences in brain images. Combat-GAM has been shown to remove unwanted sources of variability, specifically site differences, while preserving variations due to biologically-relevant covariates in the data. Harmonized data from different scanners and sites are analyzed using machine learning and deep learning methods for imaging pattern analysis. These methods integrate small yet coordinated brain effects into signatures that may yield high sensitivity and specificity in characterizing disease effects in individuals.

The dimensional approach naturally extends to other modalities, including diffusion tensor imaging, task-based functional MRI, and to molecular brain imaging with PET. We will extend these findings to studies of at-risk individuals and to clinical trials in an iterative process [30] in which the position and trajectory of a new individual in a broader dimensional system can be interrogated along with any number of clinical phenotypes, with the potential to study such cohorts over the course of illness and with treatment.

## Methods and preliminary results

To establish a NA-NF dimensional system of brain imaging biomarkers and predictors of clinical outcome, we will apply the following steps:

1. Inter-site harmonization and image analysis to create a methodological platform for constructive integration of structural imaging and neurofunctional connectivity data from multiple sites.
2. To investigate heterogeneity in neuroanatomy and functional connectivity as a collection of NA-NF patterns or dimensions, we will use semi-supervised AI methods, instead of commonly used clustering (unsupervised) approaches, in order to delineate disease-related effects, rather than variations in brain morphology and physiology that might be caused by a number of disease-irrelevant factors. Validity and relevance of identified dimensions will be verified by cross-validation and replication in independent datasets. Critically, we will derive the presence of each NA-NF signature in each participant.
3. To evaluate individual NA-NF signatures for their ability to predict or to moderate treatment response, two complementary approaches will be applied. The first approach is wholly data-driven and will assess the degree of expression of each signature to evaluate whether the position in the NA-NF dimensional system predicts treatment response. The second approach incorporates knowledge of treatment type and clinical response to enrich the NA-NF system by dimensions that are strong predictors in order to assess whether accuracy improves with the interaction of NA-NF dimension and clinical knowledge of treatment type and response.

### Participating studies and datasets

An invitation for participation was made to research groups to share ‘raw’ neuroimaging data. The present consortium provided medication-free participants with first episode or recurrent MDD, that is not treatment-resistant depression, and healthy controls.

Collaborations have been established with centers worldwide from Canada, China, European Union, United Kingdom and USA. Each center has completed a Data Sharing Agreement to provide de-identified data in accordance with institutional policies and applicable federal, state or local laws and regulations, including ethical approvals. Summary descriptions are below (Tables 1 and 2):

**Table 1:** Demographic, clinical and neuroimaging data

Clinical assessments	
Demographic data (age, sex, ethnicity, handedness, IQ, years of education)	
Standardised diagnostic criteria (DSM or ICD)	
Psychiatric history (including comorbid disorders)	
Form of depression (first episode, recurrent, treatment resistant)	
Treatment (current, history)	
Depression rating scales (HRSD, MADRS, QIDS)	
Depressive severity (mild, moderate, severe)	
Treatment outcome (baseline and post-treatment rating scale scores, remission, response)	
Neuroimaging	
Structural MRI	
Resting-state fMRI	
Diffusion tensor imaging	
PET	
EEG	
Neuropsychological assessments

**Table 2:** Preliminary study demographics, MRI field strength and MRI scanner model

Study	Number	Mean age	Age range	MRI	MRI scanner model
CAN-BIND	309	34	18–61	3 T	GE Discovery MR750
				3 T	GE Signa HDx
				3 T	Philips Intera
				3 T	Siemens TrioTim
EMBARC	336	37	18–65	3 T	GE Discovery MR750
				3 T	GE Signa HDx
				3 T	Philips Achieva
				3 T	Philips Ingenia
				3 T	Siemens TrioTim
KCL	40	30	18–45	3 T	GE Discovery MR750
LIBR	296	32	18–59	3 T	GE Discovery MR750
Manchester	70	35	20–56	1.5 T	Philips Intera
NeuroPharm1	207	28	18–59	3 T	Siemens Prisma
Oxford	70	30	19–61	3 T	Siemens TrioTim
PReDICT	344	40	18–64	3 T	Siemens TrioTim
HMRRC	269	31	18–60	3 T	GE Excite
				3 T	Siemens TrioTim
Stanford	110	33	19–58	3 T	GE Signa-Excite
STRADL	1189	60	26–84	3 T	Philips Achieva-TX
				3 T	Philips Prisma-FIT

Canadian Biomarker Integration Network in Depression (CAN-BIND) is a national depression program with recruitment from 7 centers [31, 32]. The CAN-BIND-1 treatment protocol is an 8-week trial with a selective serotonin reuptake inhibitor (SSRI) antidepressant, escitalopram, followed by an 8-week augmentation with aripiprazole if there was poor treatment response (i.e., less than 50% improvement in depressive symptoms). MRI scans were acquired at baseline, weeks 2 and 8 in both MDD and healthy participants.

Copenhagen University (NeuroPharm1) cohort consists of data from the Center for Integrated Molecular Brain Imaging (Cimbi) and Center for Experimental Medicine Neuropharmacology prospective longitudinal treatment study with an SSRI, escitalopram. The protocol is a 12-week trial with a SSRI, escitalopram, which could be switched to a serotonin norepinephrine reuptake inhibitor, duloxetine at week 4 if there were unacceptable side effects or poor treatment response (i.e., less than 25% improvement in depressive symptoms). MRI scans were acquired at baseline in healthy participants and at baseline and week 8 in MDD [33–39].

Establishing Moderators and Biosignatures of Antidepressant Response in Clinical Care (EMBARC) is a multisite, randomized, placebo-controlled clinical trial with recruitment from 4 centers [40]. Treatment protocol consists of two stages. Stage 1 is an 8-week, double-blind, randomized allocation to placebo medication or to an SSRI, sertraline, followed by Stage 2, an 8-week, double-blind, cross over treatment design. At Stage 2, participants continue treatment for 8 weeks (either placebo or sertraline) if the Clinical Global Improvement scale (CGI) rating is at least “much improved”. If the CGI rating is less than “much improved”, then treatment is switched under double-blind conditions. From the initial placebo treatment arm, treatment is switched to the SSRI, sertraline, and from the initial SSRI, sertraline, treatment arm, the treatment is switched to the non-serotonergic antidepressant, bupropion. MRI scans were acquired in medication-free MDD and healthy participants at baseline.

Huaxi MR Research Center (HMRRC) cohort consists of medication-naïve first episode MDD and matched healthy participants with single session MRI scans [41–43].

King’s College London cohort consists of 4 studies [44–50]. Treatment protocol was an 8-week trial of serotonin-norepinephrine reuptake inhibitor (SNRI), duloxetine. MRI scans were acquired at baseline, weeks 2 and 8 in both MDD and healthy participants [47].

Laureate Institute for Brain Research (LIBR) cohort consists of 2 studies with MRI data in first episode and recurrent MDD and matched healthy controls [51–53].

University of Manchester cohort consists of 3 studies [54–56]. Treatment protocol was an 8-week trial of SSRI, citalopram, with MRI scans acquired at baseline and week 8 in both MDD and healthy participants [54].

University of Oxford cohort consists of an 6-week trial of SSRI, escitalopram, with MRI scans acquired at baseline and week 6 [57, 58].

Predictors of Remission in Depression to Individual and Combined Treatments (PReDICT) study is a 12-week randomized clinical trial of treatment-naïve MDD with 3 treatment arms: an SSRI, escitalopram; an SNRI, duloxetine; or CBT, and a 12-week second phase if remission was not achieved with monotherapy, with the addition of CBT to the medication treatment arms or augmentation with escitalopram to the CBT treatment arm. MRI scans were acquired at baseline and week 12 [59, 60].

REST-meta-MDD study consists of resting state fMRI data in medication-naïve first episode and recurrent MDD from 17 sites in China [61, 62].

Southwest University (SWU) cohort consists of a community-based recruitment which includes first episode and recurrent MDD and healthy control participants [63–65].

Stanford University cohort consists of MRI data in first episode and recurrent MDD and healthy control participants [66–68].

STratifying Resilience and Depression Longitudinally (STRADL) is a community-based cohort from the Generation Scotland Scottish Family Health Study with detailed clinical, cognitive and neuroimaging assessments. Single session MRI scans were acquired [24, 69].

University of California at San Francisco (UCSF) cohort consists of first episode and recurrent MDD and healthy control participants [70, 71].

### Assessments

MDD diagnosis is based on standardised diagnostic criteria, DSM or ICD: DSM-IV (HMRRC, Manchester, Oxford, PReDICT, SWU), DSM-IV-TR (CAN-BIND, EMBARC, KCL, LIBR, Stanford, STRADL) and DSM-5 (Neuropharm1). Structured clinical interview assessments were performed: Structured Clinical Interview for DSM (SCID) (EMBARC, HMMRC, KCL, LIBR, Manchester, Oxford, PReDICT, Stanford, SWU, STRADL) or Mini International Neuropsychiatric Interview (MINI) (CAN-BIND, KCL, Neuropharm1).

Symptom severity has been measured using standardised clinician-rated symptom scales: 17-item Hamilton Rating Scale for Depression (HRSD) [72] (EMBARC, HMRRC, KCL, LIBR, Neuropharm1, Oxford, Stanford, SWU), 24-item HRSD (PReDICT), Montgomery-Åsberg Depression Rating Scale (MADRS) [73] (CAN-BIND, LIBR, Manchester; PReDICT), and Quick Inventory for Depressive Symptomatology (QIDS-SR) [74, 75] (EMBARC, PReDICT, STRADL).

Clinical measures include illness history (e.g., age onset, number of previous episodes) and medication records. Demographic information (e.g., years of education) and neuropsychological assessments, including memory and executive function, where available are included (CAN-BIND, EMBARC, KCL, LIBR, Manchester, Neuropharm1, PReDICT, STRADL).

Prospective longitudinal treatment studies have provided clinical outcome data (CAN-BIND, EMBARC, KCL, Manchester, Neuropharm1, Oxford, PReDICT). We have additional information in MDD participants taking antidepressant medication (CAN-BIND, KCL, Manchester, Neuropharm1, Oxford, PReDICT, Stanford, STRADL, SWU).

### Clinical data harmonization

High reliability, internal consistency and correspondence have been demonstrated for HRSD [76], between HRSD and QIDS [77, 78], HRSD and MADRS [79], and QIDS and MADRS [80] in outpatient non-psychotic MDD. Standardized conversions will be applied to generate comparable scores [77, 78, 81]. Clinical remission is defined as 17-item HRSD score of less than or equal to 7 and the equivalent in MADRS and QIDS, and treatment response is defined as having an improvement of at least 50% relative to baseline depressive severity [8].

It is possible that derived NA-NF dimensions could be transdiagnostic. We will also incorporate clinical knowledge using a Research Domain Criteria (RDoC)-informed approach to specify transdiagnostic dimensions, using individual items as well as symptom clusters scores from rating scales [82–85]. For example, within the Arousal and Regulatory System domain, we can specify fine-grained disturbances in sleep (i.e., initial, middle and late insomnia, as well as hypersomnia) and vegetative disturbances (i.e., appetite increase or decrease, weight increase or decrease), and in the Negative Valence Systems domain, a dimension based on items assessing anxiety reflecting sensitivity to potential threat.

While all patients have a diagnosis based on standardised criteria, we cannot rule out the possibility of site differences reflecting true cultural or ethnic differences in MDD phenomenology. Potential systemic differences in symptom profiles across sites due to different socio-cultural contexts will be examined. Common factor structures have been demonstrated across cultures [86, 87]. Principal component analyses will be performed to examine factor structures that characterize symptom profiles by site. We will control for any site differences in the magnitude of symptoms by deriving within-center standardized scores using the residuals. If necessary, we will apply factorization methods to the standardized scores to obtain illness dimensions, which we will validate by analyzing their associations with demographic, diagnostic, neurocognitive and other clinical data. We will empirically test the brain-to-symptom mappings by altering the balance between inter-site pooling and within-site normalization.

### MRI imaging characteristics

MRI data include structural MPRAGE or equivalent structural MRI scans and resting state functional MRI scans acquired on 1.5 Tesla (T) or 3 T MRI systems. Different models of scanner have been used, including Discovery MR750 3 T (GE Healthcare, Little Chalfont, Buckinghamshire, UK), Signa HDxt 3 T (GE Healthcare, Little Chalfont, Buckinghamshire, UK), MAGNETOM TrioTim or MAGNETOM Prisma Fit (Siemens Healthcare, Erlangen, Germany), and Achieva 3 T (Philips Healthcare, Best, Netherlands) (Table 2).

### MRI data integration and harmonization

Harmonization is a critical process as it enables the constructive pooling and integration of all datasets within the consortium. Although imaging protocols among all included studies are comparable, scanner and minor acquisition protocol variations nonetheless introduce inter-study differences in imaging characteristics which render direct pooling of the data very difficult. In the present consortium, we have harmonized image processing pipelines, statistical harmonization of derived measures, and deep learning based harmonization of raw images [88].

#### Image-level extraction of structural anatomy and functional connective measures

Imaging variables are extracted from the MRI scans to provide a multi-scale representation of structural and connectomic characteristics. Some features are defined a priori (e.g., multi atlas-based parcellations) and some are data-driven (e.g., structural covariance and functional connectivity networks).

##### MUSE region of interest segmentation

Volumetric MRI features are extracted via established and validated methods using a fully automated processing pipeline. Raw 3D T1-weighted MRIs are first quality checked for motion, image artifacts, or restricted field-of-view. Each participant’s quality-controlled T1-weighted MRI scan is preprocessed with a containerized processing pipeline. Preprocessing steps consist of magnetic field intensity inhomogeneity correction [89] and multi-atlas skull-stripping for the removal of extra-cranial material [90].

The images are segmented using a state-of-the-art, multi-atlas, multi-warp label-fusion method, MUSE [91]. In this framework, multiple atlases with semi-automatically extracted ground-truth region of interest (ROI) labels are first warped individually to the target image using two different non-linear registration methods. A spatially adaptive weighted voting strategy is then applied to fuse the ensemble into a final segmentation. This procedure is used to segment each image into 145 anatomical regions of interest (ROIs) spanning the entire brain. We calculate the volumes of the 145 ROIs, as well as the volumes of 113 composite ROIs that are obtained by combining individual ROIs into larger anatomical regions following a predefined ROI hierarchy whereby brain anatomy can be quantified at multiple levels of resolutions [91].

MUSE has obtained top accuracy in comparison with multiple benchmark methods in independent evaluations [92]. MUSE utilizes state-of-the-art multi-atlas, multi-warp algorithms, with a very rich set of atlases spanning several protocols from 1.5 T SPGR images to 3 T MPRAGE images. The ensemble of atlases used to segment each scan contains sufficient diversity which renders it robust to scanner variations [91]. Critically, we have leveraged several studies which have scanned the same individuals in multiple scanners and hence have built procedures that render the multi-scanner atlases mutually consistent [91].

Voxel-based, tissue-specific (grey matter, white matter, cerebrospinal fluid) regional volumetric maps are obtained using MUSE segmentation and inverse atlas warping is applied for voxel-based analyses. Voxel-wise regional volumetric maps (RAVENS) are generated for each tissue [93] by spatially aligning the skull-stripped images to a template in MNI-space [94]. Quality control (QC) on derived maps and imaging variables is performed using a semi-automated approach. An automated procedure automatically ranks scans based on outlying values of quantified metrics (i.e., ROI values) and flags those that show deviation from estimated population distributions. Flagged images are examined by visual inspection using a visualization and annotation tool for evaluating for pipeline failures (e.g., poor brain extraction, tissue segmentation, and registration errors).

##### Structural covariance networks

Our prior work has highlighted the potential of structural covariance networks as a flexible and biologically interpretable way of reducing complex anatomical images down to a relatively small set of measurements [95, 96]. This approach uses orthogonally-projective non-negative matrix factorizations to parcellate the brain into regions that show consistent trends across individuals, potentially because they are influenced by common underlying neurobiological factors. For example, we found that patterns of structural covariance were highly reproducible, aligned well with functional networks, displayed differential developmental trajectories during adolescence, and correlated with maps of evolutionary cortical expansion [96]. We have recently developed a novel method that computes multi-scale non-negative matrix factorization (NMF) components [97]. This allows us to characterize heterogeneous presentation of neuropsychiatric and neurodegenerative diseases in multiple scales. These networks will be part of our imaging feature panel and might be well-suited for capturing the neurodevelopmental underpinnings of MDD.

##### Deep learning generative adversarial networks (GAN)

**Fig. 1:**
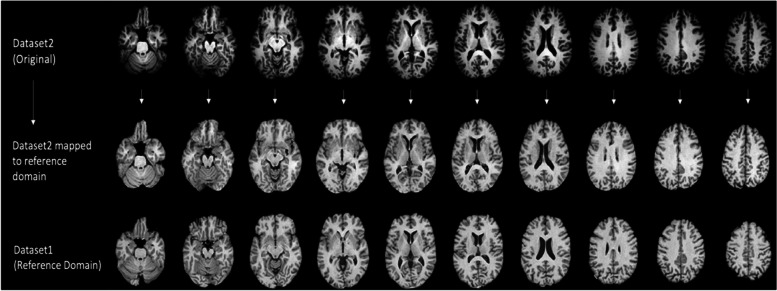
Example of image harmonization via deep learning based canonical mapping to a reference domain presented in bottom row

A second approach utilizes deep learning CycleGAN and STAR-GAN methods [88], which synthesize images drawn from certain distributions. This method has been used extensively in numerous applications seeking to transform images from one style to another. We have used data from numerous scanners to learn the mapping of brain MRI scans to a canonical reference domain, in which inter-scanner variations are minimized [88] (Fig. 1). By making the characteristics of images very similar, while preserving important information, subsequent image analysis steps, including segmentation and parcellation, become significantly more consistent across studies. We will further refine and adapt this method to this project, including some of the current directions that involve nonlinear modelling co-variates in order to better restrict these mappings to differences related to MRI scanners and protocols, while preserving anatomical characteristics.

##### Personalized functional networks

**Fig. 2:**
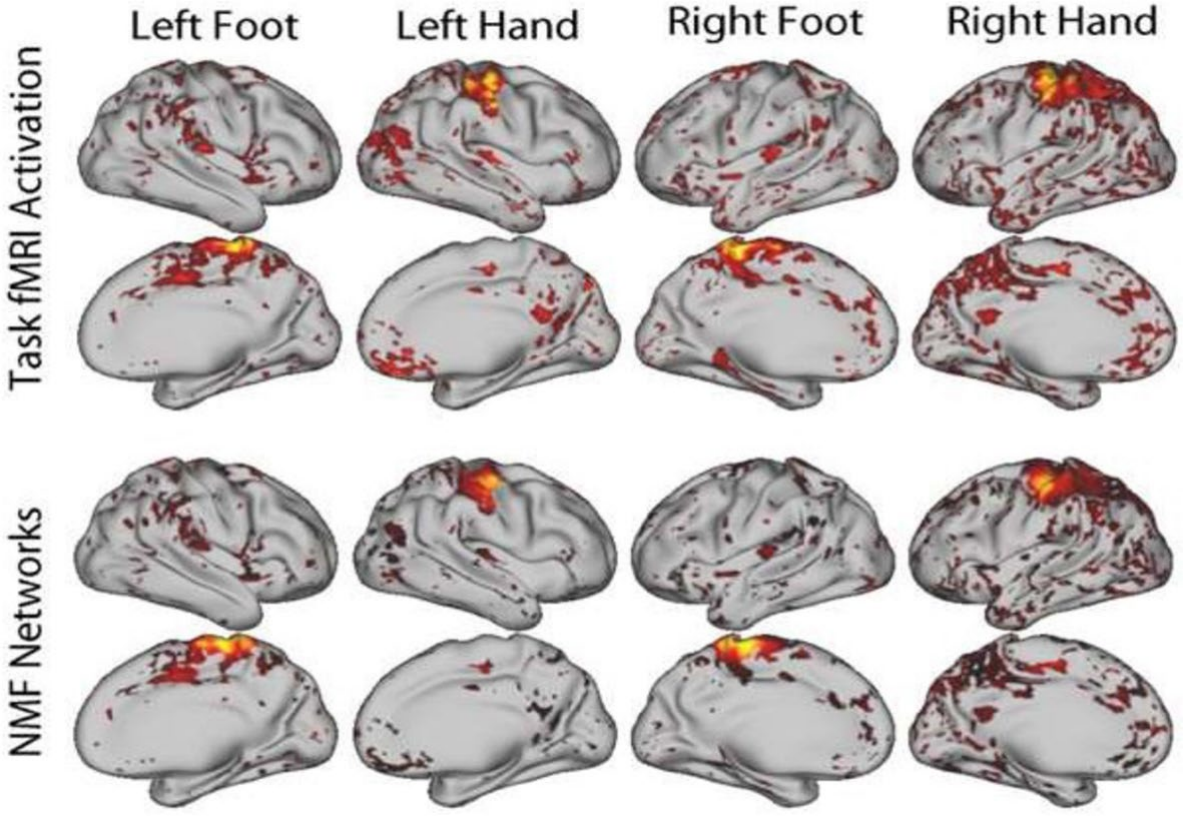
Personalized functional networks defined using NMF with resting state fMRI data aligned with activation during a motor task, adapted from Li et al. [98]

**Fig. 3:**
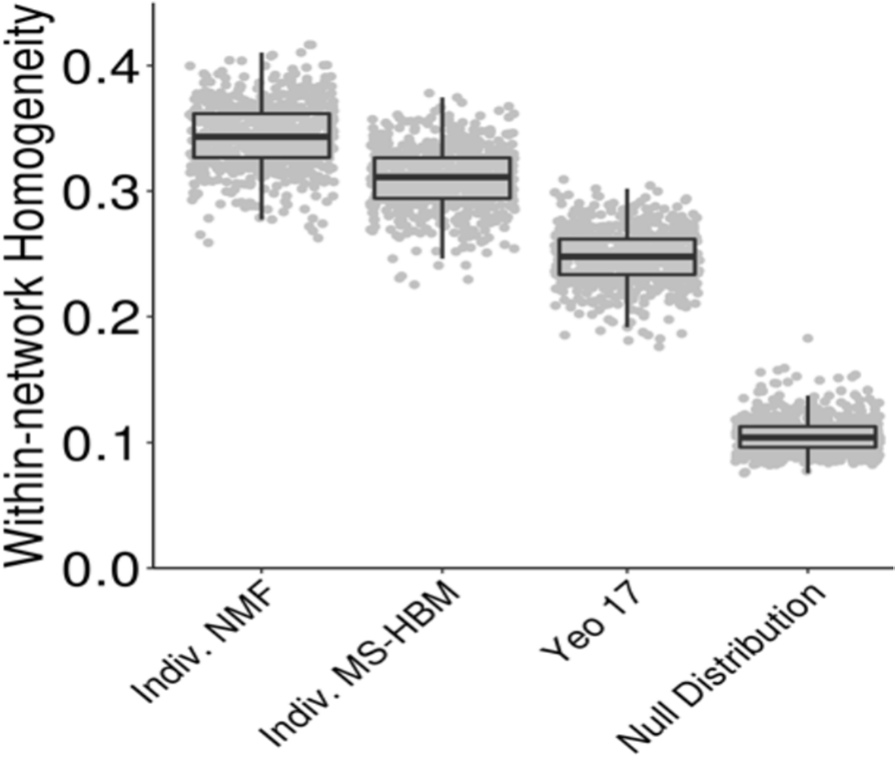
Personalized functional networks improve functional homogeneity. Functional homogeneity is higher within personalized functional networks (from either non-negative matrix factorization (NMF) or multi-session hierarchical Bayesian model (MS-HBM)) than standard group-level networks (Yeo 17-network group atlas (Yeo 17)) or a null model that preserves spatial covariance structure, adapted from Cui et al. [105]

For resting state fMRI, personalized functional networks using a spatially-regularized NMF method align well with functional activation patterns and have improved functional homogeneity [98] (Fig. 2). Functional homogeneity quantifies the degree to which a functional network represents one signal, rather than mixing multiple neurophysiological signals. Measures of functional connectivity, derived from brain parcellations and functional networks show promise in predicting brain maturity and distinguishing disease from healthy brain states [99–102]. Furthermore, there are significant correlations between elements of the connectivity matrices, which may lead to unreliable classification [103]. Using our NMF method, we found increased homogeneity compared to standard network definitions using a group atlas, as well as a null distribution created using a conservative spin-based spatial permutation procedure [104]. The personalized functional networks delineated from our NMF method showed that the networks with the greatest variability in functional topography in youth are the very higher-order association networks impacted by psychopathology, these association networks are refined with age in development, and individual differences in association network topography predicted executive function [104] (Fig. 3).

**Fig. 4:**
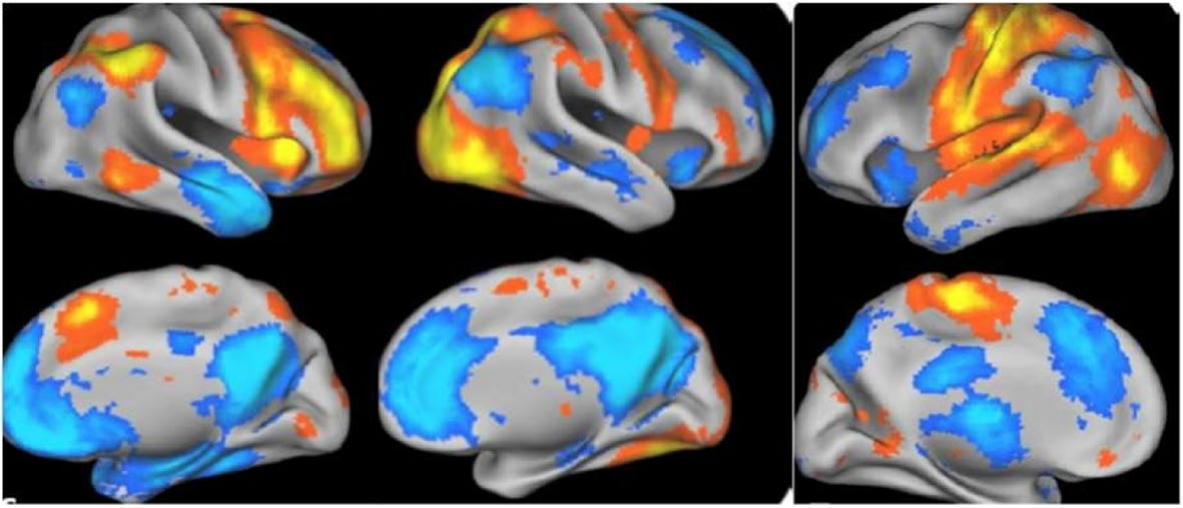
Three representative SCPs identified. Left column: Default mode anti-correlated with fronto-parietal network; Middle column: visual and default mode anti-correlated regions; Right column: sensorimotor regions anti-correlated with fronto-parietal network; blue and orange colours represent anti-correlated regions, adapted from Eavani et al. [109]

We are also employing a complementary method based on sparse connectivity pattern (SCP) learning, leveraging the effective non-linearity of sparse dictionary learning [104, 106–108] as a means for describing the functional connectivity patterns of brain networks [109]. SCPs also minimize the negative impact of correlated features on the robustness of prediction models [103] (Fig. 4). A hierarchical extension of this approach extracts these functional networks at multiple scales [110]. Adopting these complementary techniques will provide a rich set of imaging features to characterize the functional brain connectome.

### Harmonization of derived measures

**Fig. 5:**
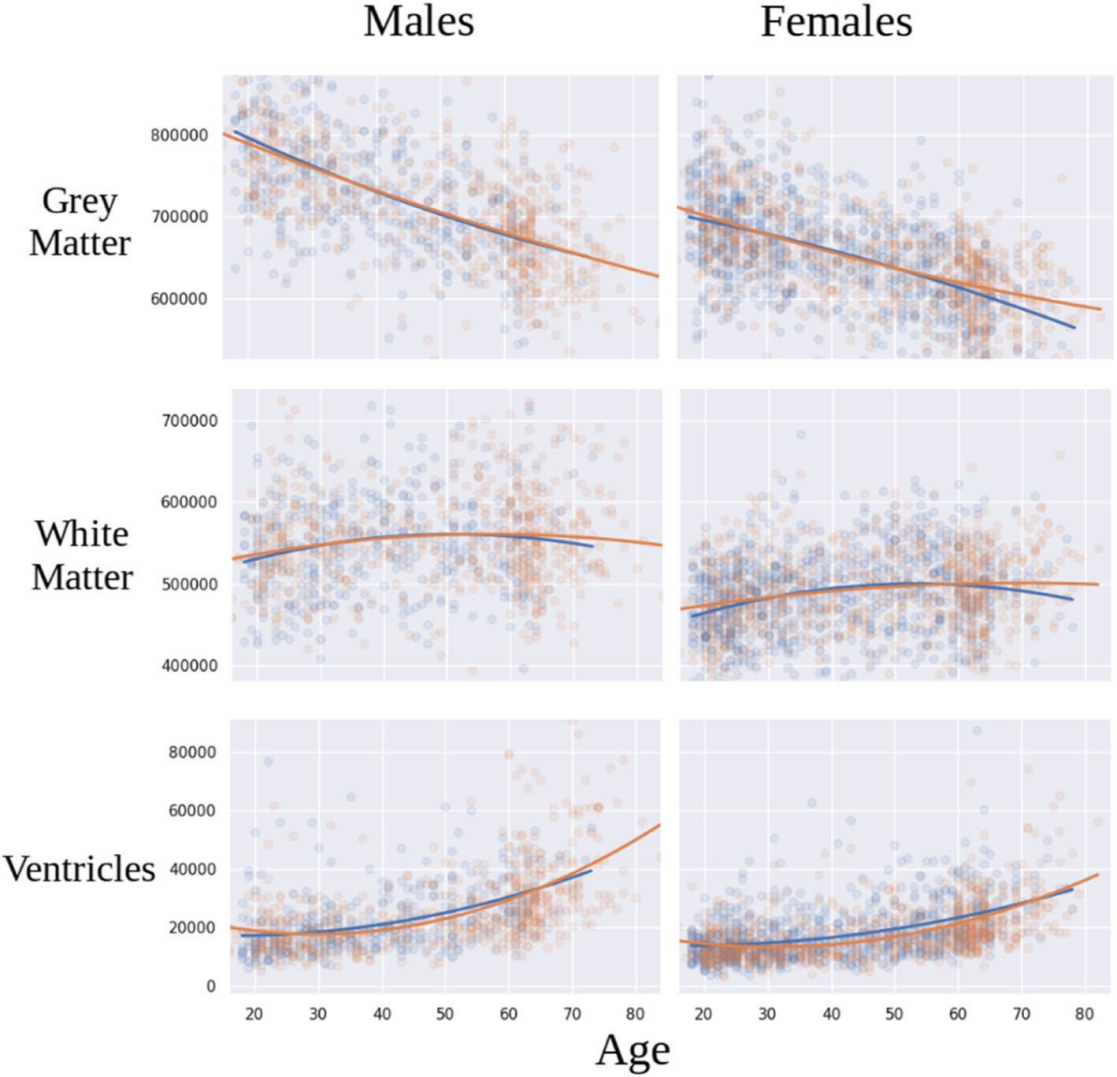
Preliminary results from the COORDINATE MDD datasets (EMBARC, Oxford, HMRRC, Stanford, STRADL) showing age trajectories in grey matter, white matter and ventricular volumes in MDD patients (colored blue) as compared to healthy controls (colored red)

We have developed a statistical harmonization approach [29], building upon the COMBAT method that has been successfully used for over a decade to remove batch effects in genomic studies and recently adapted in neuroimaging research [111–114]. This approach is fully multivariate, utilizing hyper-parameters to define over-arching statistical priors, and has been successfully adopted in imaging. In order to model nonlinear effects of covariates (e.g., age), we have combined COMBAT with generalized additive models (GAMs) using spline functions. The resultant COMBAT-GAM general tool for harmonization can be applied to various forms of data, including ROIs and coefficients of structural covariance and functional connectivity networks. Preliminary results of statistical harmonization from the present consortium are shown in Fig. 5.

**Fig. 6:**
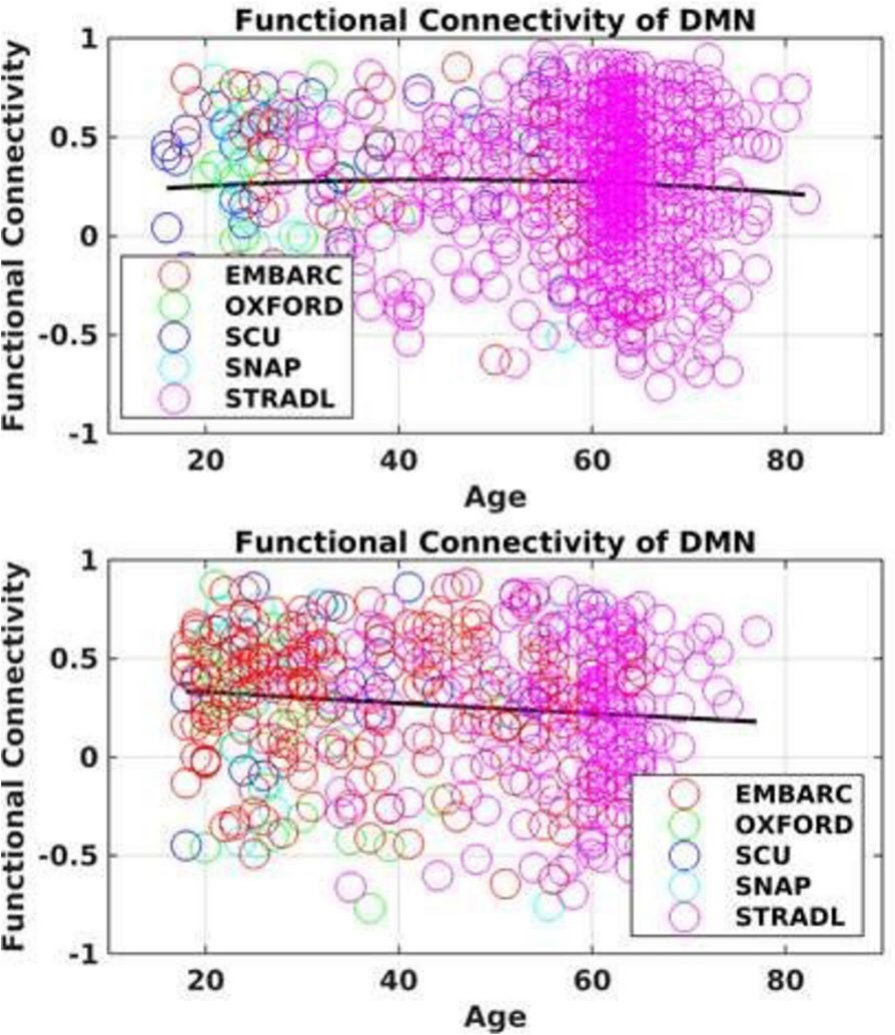
Preliminary results showing functional connectivity of DMN (between anterior cingulate cortex and posterior cingulate cortex) in healthy controls (top panel) and MDD participants (bottom panel) from the COORDINATE MDD datasets (EMBARC, Oxford, SCU (HMRRC), SNAP (Stanford), STRADL)

Harmonization of resting state fMRI data is typically performed at the correlation matrix level. In particular, we have developed a functional connectivity covariance batch effect correction (FC-CovBAT) [115] that models second order moments of the upper-triangular elements of individual correlation matrices derived from the fMRI data. FC-CovBAT is an extension of COMBAT and CovBat methods [116] for structural imaging data. These methods statistically model the site/scanner differences not only in the means and variances of the multivariate correlation values, but also in the covariance structures between the multivariate correlation values from the FC data (Fig. 6).

### NA-NF dimensional neurobiological representation of MDD

**Fig. 7:**
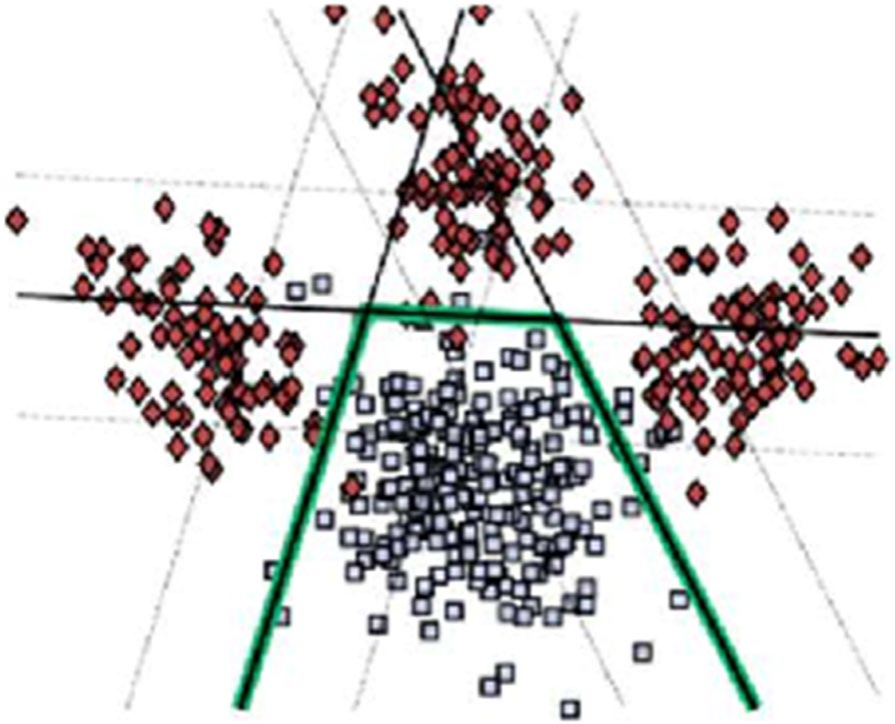
Healthy controls (green) are separated from patients via a number of maximum-margin hyperplanes that define disease subtypes. Iterative classification and clustering determine subtype membership and classification hyperplanes

Direct clustering of patient data, especially high-dimensional images, is challenged by effects of confounding variations in the data, namely variations in healthy individuals that are not related to disease effects. We have developed semi-supervised clustering methods to account for these effects, effectively clustering differences between patients and healthy controls, rather than directly clustering patient data. The methods assume that patient data have been derived from healthy control data via one of several, to be estimated, transformations that reflect disease effects (Fig. 7). Whatever variations are present in the healthy control data and are unrelated to disease effects will follow the disease-specific transformations. Within this approach, we acknowledge that some control samples might reflect a “super-healthy” control group without any chronic diseases that may represent resilience [117], though healthy control participants have also been recruited from the general community in the present consortium.

Two semi-supervised methods will be used: 1) HYDRA, a conventional machine learning approach that is largely a discriminative method [16], with its multi-scale extension [118]; and 2) Generative Adversarial Networks (GANs), a complementary method which leverages the power of a class of generative deep learning methods [119]. These methods have complementary strengths, which will be combined in seeking robust and reproducible MDD dimensions.

#### HYDRA

HYDRA uses the concept of a convex polytope, in conjunction with support vector machine principles (maximum margin cost function), to simultaneously classify patients from healthy controls and to determine a number of hyperplanes that represent disease dimensions. An iterative process determines the disease dimensions, as well as memberships in them. HYDRA has recently helped discover two distinct neuroanatomical dimensions in schizophrenia with evidence of differences in cognitive and clinical profiles between dimensions, in polygenic risk scores for schizophrenia and autism, as well as in brain development and aging studies [120]. We will apply this method to the imaging feature panels (ROI volumes, structural covariance network coefficients derived from voxel-based maps, and functional connectivity network coefficients), in order to identify MDD dimensions and to obtain positions of each patient within each dimension. It is likely that some patients will express multiple NA-NF signatures concurrently.

#### Deep learning and generative adversarial networks (GANs)

**Fig. 8:**
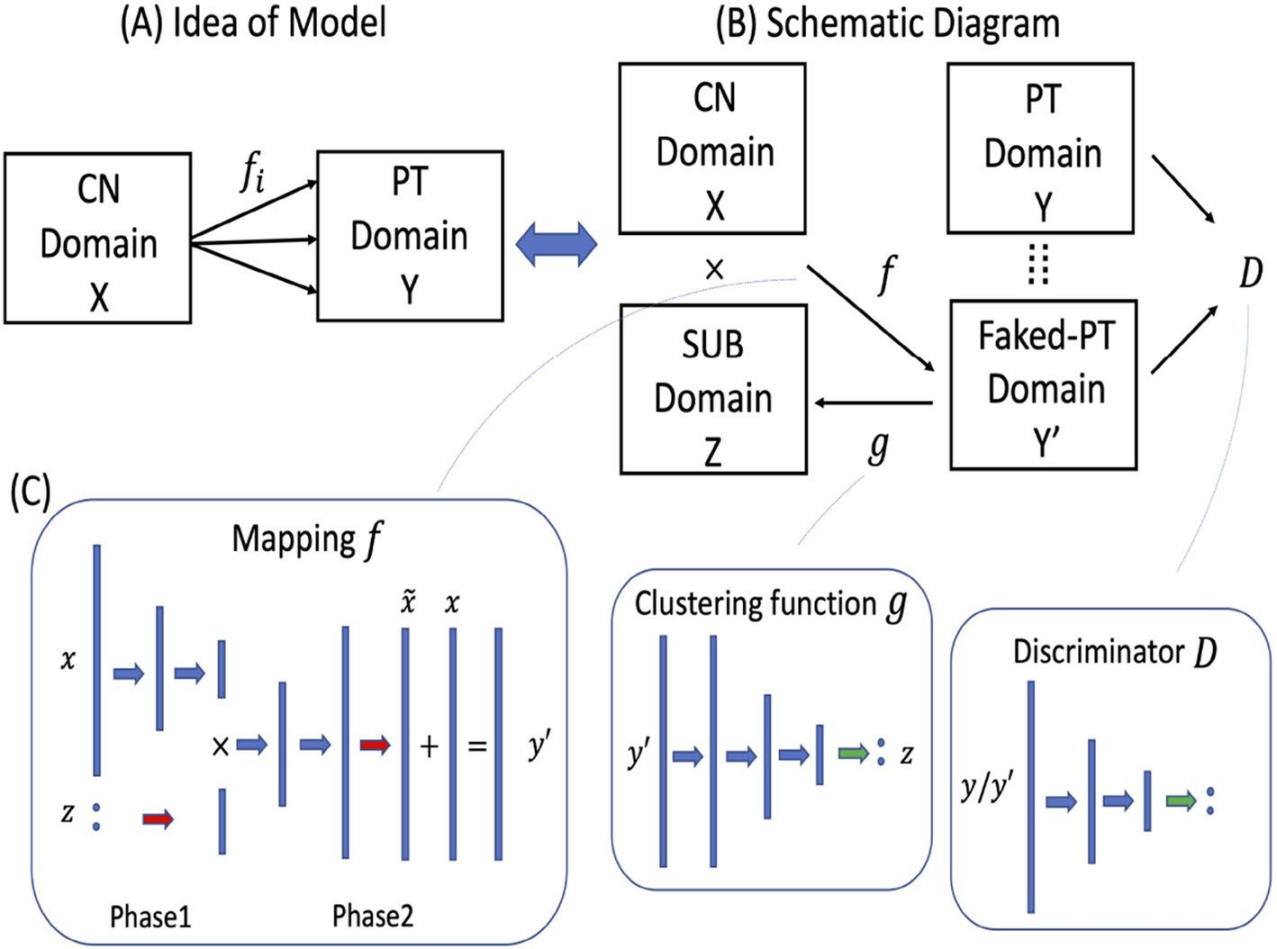
Our deep learning-based approach to subtyping. **A** General idea behind Smile-GANs (**B**) Schematic diagram of Smile-GANs. CN: healthy control, PT: patient, SUB: subtype (**C**) Network architectures of the three main functions (f, g, D): blue arrow represents one linear transformation followed by one leaky relu function, green arrow represents one linear transformation followed by one softmax function, red arrow represents only one linear transformation. The GAN network learns to synthesize MDD patient scans from scans of normal controls, contingent upon subtype, SUB, which is learned in the training process. The discriminator, D, of this GAN ensures that these synthesized scans are indistinguishable from real MDD patients scans. The mappings, f, estimated in this process capture features of the neuropathologic processes that transform imaging data of controls to those of MDD patients, adapted from Yang et al. [119]

The second approach relies on a generative methodology for semi-supervised clustering, termed Smile-GAN, which utilizes state-of-the-art deep learning CycleGAN architectures, with latent variables representing neurobiological dimensions [119]. The model simultaneously learns the mapping and clustering functions using the discriminator function and the data from the patient domain. Representations of the patient clustering structure and the depression-related NA-NF dimensions are stored respectively in the network weights of functions after learning, which are the main outcomes of the model training (Fig. 8).

While these approaches can be very powerful, they require large training datasets. The availability of a sufficiently large and well-harmonized dataset in the present consortium will allow us to harness the power of these deep learning methods. We will seek experimental confirmation via split sample and sensitivity analyses.

We use cross-validation, split-sample, and random permutation experiments to determine the optimal number of reproducible and statistically significant clusters corresponding to NA-NF dimensions of MDD, by measuring the adjusted rand index (ARI) [120]. The most reproducible clusters, according to two main criteria, both high and statistically significant ARI, will be used to derive the NA-NF dimensions. As part of the method, cluster memberships of each individual will be readily calculated [16, 119]. These memberships will effectively be the coordinates of an individual in the NA-NF coordinate system, reflecting the dominant NA-NF MDD patterns.

##### Incorporating clinical knowledge

We will augment this approach with the use of clinical measures, namely symptom profile as well as depressive state and medication status. The main idea is that NA-NF dimensions might not necessarily be easily interpretable clinically. By incorporating clinical phenotypic measures in parts of the clustering procedure, we will favor imaging patterns that simultaneously have relatively distinct clinical phenotypes. Methodological extensions will include, for example, introducing a discriminant term, which encodes the dimensions. This term will encourage NA-NF dimensions that have relatively distinct clinical phenotypes. We will also modify the latent space formulation to allow for continuous latent variables, in order to better model anatomy and function in individuals who might present mixed patterns.

### Multidimensional predictors of treatment responses

A main goal is to investigate whether position in the NA-NF dimensional system captures disease effect in predicting treatment response at the individual level based on semi-supervised approaches. Treatment response will be defined as the harmonized change in clinician-rated depressive severity scales. Changes of scores after the treatment period will be modelled both as continuous values and binary variables. We recognise that these measures are often insufficient for capturing treatment efficacy and might not reflect patient experience. We will further investigate whether NA-NF dimensions might map onto subfacets of the scales with greater clinimetric properties [121].

We will further enrich NA-NF dimensions by directly and specifically looking for patterns that predict treatment response via supervised classification. Such imaging signatures might or might not capture broad and treatment-oblivious NA-NF variations, however they capture NA-NF measures that are tailored to prediction of treatment response in which baseline clinical variables will be part of these treatment-specific predictive models.

In particular, specificity to particular categories of treatment will be investigated. Prediction of differential response between placebo and antidepressant medication is highly important, yet difficult to assess. Prediction of placebo response is uniquely accessible in the present sample, in which treatment outcome data are available for both active antidepressant and placebo arms of randomized controlled trials.

To identify pre-treatment moderators of differential treatment response, the specificity of NA-NF predictors to different categories of antidepressant medication will be investigated. Treatment outcome data are available for SSRI and SNRI medication classes. Specificity of NA-NF predictors of response to psychotherapy or to antidepressant medication will also be investigated.

A key weakness of multivariate pattern analysis studies has been the frequent lack of generalizability and validation on new datasets. Given the size of the consortium, we will test the NA-NF dimensional system in wholly independent prospective longitudinal treatment cohorts, namely Copenhagen University. We emphasize that cross-validation methods are engrained in all our analyses to reduce overfitting and improve generalizability.

## Summary

COORDINATE-MDD brings together deeply phenotyped clinical data, multi-site ‘raw’ individual-level structural and functional neuroimaging data, and state-of-the-art AI-based methods in order to identify the multimodal dimensions that comprise MDD and which inform treatment response. Our consortium data are derived from adults with first episode or recurrent MDD, medication-free, in a current major depressive episode, that is not treatment-resistant depression, and healthy controls. We leverage statistical harmonization, machine learning and deep learning methods and large integrated and harmonized sample of highly-phenotyped, individual-level patient data with prospective longitudinal treatment outcomes. Our objectives are to delineate the robust and reproducible neurobiological NA-NF signatures which comprise MDD and which predict or moderate treatment response. We will follow an iterative process by first defining the multivariate neural dimensions that characterise deeply phenotyped samples and then testing the utility of these dimensions in novel samples.

## Data Availability

Original data can be requested from each consortium member. Derived data in the present consortium can be made available from the corresponding author on reasonable request and with permission of each consortium member.

## Abbreviations

MDD: Major depressive disorder
NA-NF: Neuroanatomical-neurofunctional
SSRI: Selective serotonin reuptake inhibitor
CAN-BIND: Canadian Biomarker Integration Network in Depression
Cimbi: Center for Integrated Molecular Brain Imaging
EMBARC: Establishing Moderators and Biosignatures of Antidepressant Response in Clinical Care
CGI: Clinical Global Improvement scale
HMRRC: Huaxi MR Research Center
SNRI: Serotonin-norepinephrine reuptake inhibitor
LIBR: Laureate Institute for Brain Research
PReDICT: Predictors of Remission in Depression to Individual and Combined Treatments
SWU: Southwest University
STRADL: STratifying Resilience and Depression Longitudinally
UCSF: University of California at San Francisco
MINI: Mini International Neuropsychiatric Interview
HRSD: Hamilton Rating Scale for Depression
MADRS: Montgomery-Åsberg Depression Rating Scale
QIDS-SR: Quick Inventory for Depressive Symptomatology
RDoC: Research Domain Criteria
T: Tesla
ROIs: Regions of interest
GAN: Generative adversarial networks
NMF: Non-negative matrix factorization
SCP: Sparse connectivity pattern
GAMs: Generalized additive models
FC-CovBAT: Functional connectivity covariance batch effect correction
GANs: Generative Adversarial Networks
ARI: Adjusted rand index
